# Long noncoding RNA in cardiac aging and disease

**DOI:** 10.1093/jmcb/mjz046

**Published:** 2019-06-01

**Authors:** Noelia Lozano-Vidal, Diewertje I Bink, Reinier A Boon

**Affiliations:** 1 Department of Physiology, Amsterdam Cardiovascular Sciences, Amsterdam Universitair Medische Centra, Vrije Universiteit Amsterdam, 1081 HZ, Amsterdam, the Netherlands; 2 Institute of Cardiovascular Regeneration, Goethe University, Frankfurt am Main, Germany; 3 German Center for Cardiovascular Research (DZHK), Partner Site Rhein-Main, Berlin, Germany

**Keywords:** long noncoding RNA, aging, cardiovascular disease

## Abstract

Cardiovascular diseases (CVDs) are the main cause of morbidity and mortality in Western society and present an important age-related risk. With the constant rise in life expectancy, prevalence of CVD in the population will likely increase further. New therapies, especially in the elderly, are needed to combat CVD. This review is focused on the role of long noncoding RNA (lncRNA) in CVD. RNA sequencing experiments in the past decade showed that most RNA does not code for protein, but many RNAs function as ncRNA. Here, we summarize the recent findings of lncRNA regulation in the diseased heart. The potential use of these RNAs as biomarkers of cardiac disease prediction is also discussed.

## Introduction

Age-related diseases have become a new health care challenge to face in Western countries due to the progressive aging of the population. With the increasing life expectancy, it is estimated that most of the babies born after the year 2000 will live up to their 100th birthday (Christensen et al., 2009). Although the efforts to improve the outcome of circulatory diseases have led to a decrease in the mortality associated to them (Malvezzi et al., 2018), these are still the leading cause of death worldwide, and their prevalence is greater in the elderly (Kovacic et al., 2011a; Vigen et al., 2012; Gaeta et al., 2017). Therefore, aging is the principal risk factor for cardiovascular diseases (CVDs), and it is a burden for healing after myocardial infarction (Wellenius and Mittleman, 2008; North and Sinclair, 2012), which will have a great impact in health care costs (Heidenreich et al., 2011).

Transcriptomic data revealed that the expected scenario of gene expression was wrong. Protein-coding genes account for <3% of the transcriptome (Okazaki et al., 2002). The rest are noncoding RNAs (ncRNAs), divided in two classes according to their length, small (including miRNAs, tRNAs, and small nucleolar RNAs) and long ncRNAs (lncRNAs; Taft et al., 2010; Uchida and Dimmeler, 2015). According to the last version of the GENCODE project, the human genome contains 19940 protein-coding genes, 16066 lncRNA genes, and 7577 short ncRNA genes, of which 1881 are miRNAs (Frankish et al., 2019). LncRNAs are defined as transcripts longer than 200 nt without coding potential (Mercer and Mattick, 2013). They are transcribed by RNA polymerase II in relatively low levels and present little sequence conservation between species (Ulitsky et al., 2011), which complicates their detection and characterization. There are several classifications for lncRNAs depending on genomic localization and orientation (intergenic, intronic, enhancer-associated, sense, antisense, and bidirectional), their subcellular localization (nuclear or cytosolic), and their mechanism of action.

The molecular mechanisms by which lncRNAs exhibit their effects are very diverse and include epigenetic regulation, (post)transcriptional gene regulation, and compartmentalization (Maass et al., 2014). Most of these functions are carried out by binding of the lncRNA to RNA-binding proteins, such as transcription factors, chromatin modifiers, and scaffolding proteins. LncRNAs can also control the localization, interaction, and availability of effectors in a specific site to elicit a specific activity (Mao et al., 2011; Kaneko et al., 2014; Uchida and Dimmeler, 2015; Schmitt et al., 2016; Militello et al., 2018). In the past decade, increasing evidence points to a major role of lncRNAs in development and disease, including aging and CVD (Boon et al., 2016b; Frank et al., 2016; Kim et al., 2016; Hermans-Beijnsberger et al., 2018). Therefore, lncRNAs are arising as a new frontier for interventional therapy in cardiovascular aging. In this review, we will summarize the recent discoveries of the roles of lncRNAs during aging in the heart.

## Aging and senescence in the myocardium

Aging, at a cellular level, is defined by a well-described series of molecular hallmarks (Lopez-Otin et al., 2013; Paneni et al., 2017). One of them is cellular senescence, a process by which the chromosomal telomeres shorten with each cell division. While this is a physiological consequence of aging, in certain situations, the eroded chromosomal endings tend to fuse, leading to chromosome instability, DNA damage, and eventually apoptosis (Calado and Young, 2009). Telomere attrition has been identified as a risk factor for several CVDs, such as myocardial infarction (Boon et al., 2013; Bar et al., 2014), atrial fibrillation (Staerk et al., 2017), and cardiomyopathy (Leri et al., 2003; Sahin et al., 2011; Sharifi-Sanjani et al., 2017). Also, an increase in mitochondrial production of reactive oxygen species during senescence further contributes to genomic instability (Kornfeld et al., 2015). Moreover, lifestyle choices contribute to the acquisition of epigenetic marks in the genome that can alter the pattern of gene expression (Kovacic et al., 2011a; Mathiyalagan et al., 2014), and aging can promote aberrant chromatin modifications (Lopez-Otin et al., 2013), influencing the development of CVDs (Greco and Condorelli, 2015).

These molecular cues of aging have a direct impact on the functionality of cells and tissues in the cardiovascular system. In summary, they promote functional changes that overlap and accumulate, such as vascular stiffness, endothelial dysfunction, altered signaling pathway activation, electrophysiological changes, and reduced proliferation (Paneni et al., 2017). At a myocardial level, the interrelation of these aging-related phenotypes will lead to the development of cardiac diseases as cardiac hypertrophy (CH), heart failure (HF) with preserved ejection fraction (HFpEF), acute myocardial infarction (AMI), and arrhythmia (AF). All of these have been found to be caused, influenced, or ameliorated by the action of lncRNAs.

### Aging-mediated CH and HF

During vascular aging, the increased stiffness of the aorta and endothelial dysfunction lead to stenosis and hypertension, provoking an increase in cardiac mass to cope with a greater demand for pumping. The result of this process is known as CH, and when it is sustained in time, remains irreversible and causes HF (Frey and Olson, 2003; Heineke and Molkentin, 2006; van Berlo et al., 2013).

HF can be classified in two types depending on the preservation of the ejection function. Therefore, we can distinguish between HF with reduced ejection function (HFrEF) or HFpEF. Typically, HFpEF is most prevalent in the elderly and its onset diagnosis is increasing as a consequence of increased aging of the population (Owan and Redfield, 2005). HFpEF is caused by a loss of the myocardium elasticity, which reduces the capability of a correct filling, and results in impaired relaxation and diastolic dysfunction (Redfield, 2016).

### Myocardial infarction is highly prevalent in the elderly

The progressive decline in vascular function together with the prolonged lifetime exposure to risk factors in the elderly creates an environment for the development for atherosclerosis, coronary heart disease, and eventually AMI (Williams et al., 2002; Lakatta and Levy, 2003a; Kovacic et al., 2011b; North and Sinclair, 2012). AMI is caused by the interruption of blood supply to a part of the heart, therefore causing hypoxia or ischemia of the cardiac muscle. Afterwards, the heart experiences a remodeling phase that often leads to HFrEF (Frangogiannis, 2015). The survival rates after AMI are also lower with advancing age (Fleg et al., 2013).

### Arrhythmia, cause and consequence of HF in the elderly

During aging, cardiomyocytes develop dysfunctional calcium handling that affects their electrophysiological properties, increasing the incidence of AF and arrhythmias in the aged population (Bootman et al., 2011; Feridooni et al., 2015). In aged patients, the increase in left atrial size is another risk factor for AF (Lakatta and Levy, 2003b).

AF and HF predispose to each other. On one side, AF abruptly changes heart rate (Saltzman, 2014), obligating the heart to increase the force of contraction and therefore promoting CH. On the other side, several aging-prevalent cardiac diseases can lead to ventricular arrhythmia by creating an arrhythmogenic environment and generating fibrotic tissue that interrupts the conduction of the electrical impulse (Frangogiannis, 2015). The comorbidity of AF and HFpEF in elderly patients results in poor clinical outcomes (Ling et al., 2016).

## Mechanism of action of lncRNAs in cardiac diseases

The mechanisms by which lncRNAs exert their function are variable and still subject of discussion. In some cases, the same lncRNA has been suggested to act in different diseases or cell types with a specific mechanism for each one. In general, most lncRNAs function by binding to ribonucleoproteins (RNPs) and modulating their functions, but there has been an increase in the identification of lncRNAs that bind to miRNAs to inhibit their interference on the expression of a third target, creating a lncRNA–miRNA–target axis. Also, some lncRNAs bind to DNA targets and attract chromatin modifier proteins, creating RNA–DNA–protein complexes to form nuclear domains (Quinodoz and Guttman, 2014).

**Figure 1:**
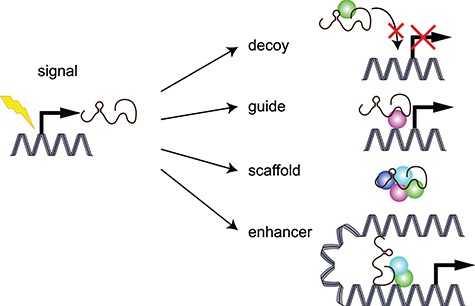
Classification of lncRNAs according to their molecular mechanism of action.

We can divide lncRNAs regarding their mechanism of action in five groups, although these mechanisms may overlap and they are not exclusive (Wang and Chang, 2011; Devaux et al., 2015; Figure 1):

(i) Signal: the expression of a lncRNA is regulated in response to a stimulus, at a certain developmental time or in a specific cell type, which helps integrating the response to that cellular context to generate quick regulatory changes. Signal lncRNAs usually perform their function toward targets through one of the mechanisms listed below.
(ii) Decoy: the lncRNA binds to an RNA-binding protein or an RNA molecule (transcription factors or other regulatory effectors) and inhibits its biological function by sequestering it. LncRNAs acting as miRNA sponges would be included in this group, and, in this case, the suggested term is competitive endogenous RNAs.
(iii) Guide: the lncRNA binds to a transcription factor or chromatin modifier and conducts it to the target promoter to alter the expression of a certain gene. This mechanism can occur in cis (on neighboring genes) or in trans (on distant genes).
(iv) Scaffold: the lncRNA binds through distinct domains to several RNA-binding proteins, bringing them together so they can interact in macromolecular complexes (RNPs).
(v) Enhancer: the lncRNA interacts with both the promoter and enhancer regions of a gene and creates a chromosomal looping to bring them in proximity and stimulates gene expression.

**Figure 2:**
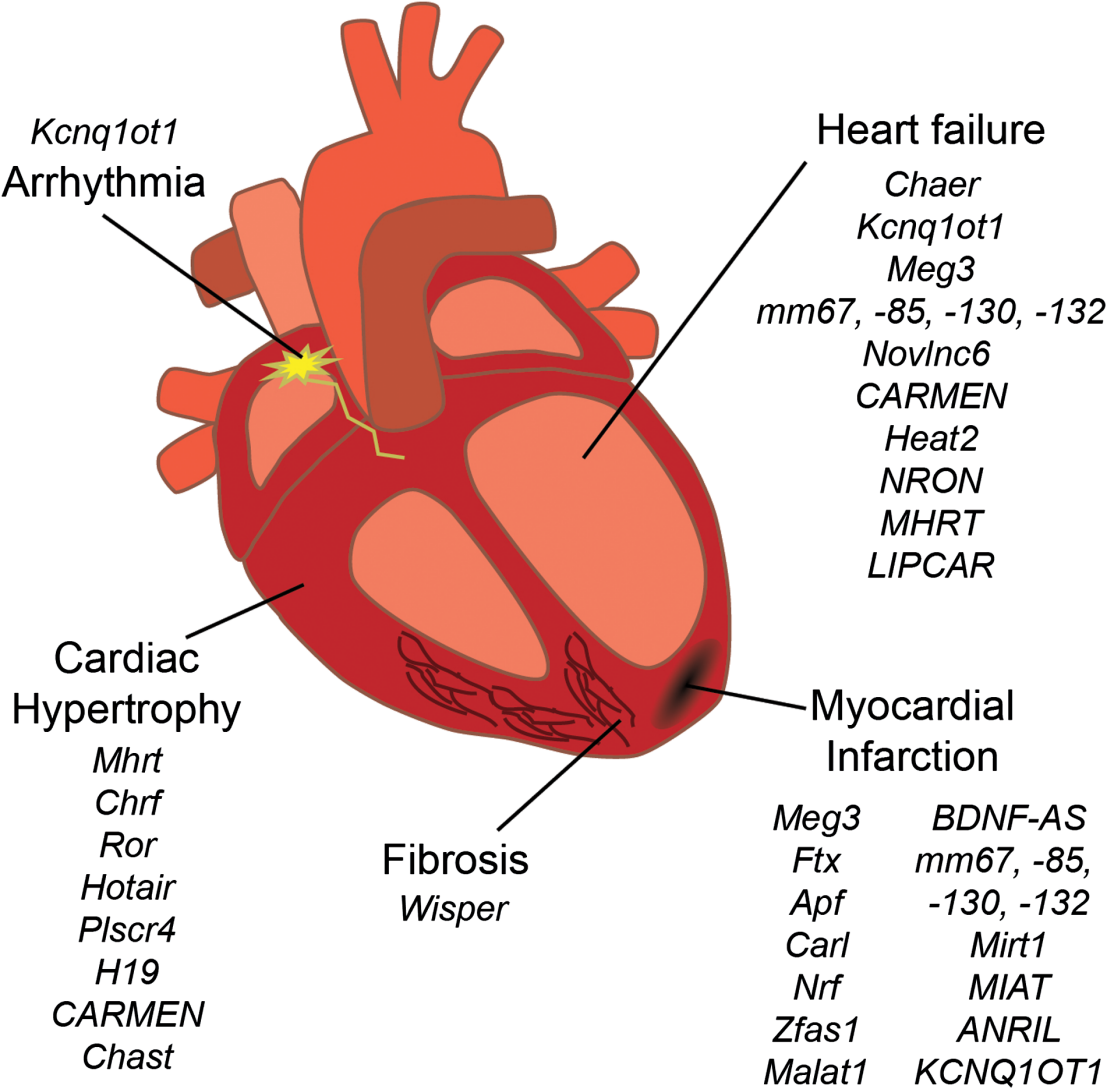
Prevalent CVDs in the aging heart and lncRNAs associated to them.

In the following sections, we will discuss the currently known lncRNAs that are involved in aging-mediated cardiac diseases attending to this mechanistic classification (Figure 2). As exception, we will omit signal lncRNAs. During the onset of a cardiac disease, the expression of a certain lncRNA is sooner or later a consequence of the disease-triggering stimulus; therefore, all of the lncRNAs reviewed here are part of this category as well as they exert their function by one of the other four.

### Decoy lncRNAs

In 2014, Han et al. (2014) showed that an antisense transcript to *Myh7*, termed myosin heavy-chain-associated RNA transcripts (*Mhrt*), was downregulated in pressure overloaded hearts and prevents the progression of cardiomyopathy. *Mhrt* originates antisense to the intergenic region between *Myh6* and *Myh7* and was found to mediate the expression switch from *Myh6* to the embryonic *Myh7*, a hallmark of CH (Miyata et al., 2000), by binding to *Brg1* in a *cis*-regulated, chromatin modifying feedback circuit (Han and Chang, 2015). Also in CH, cardiac-hypertrophy-associated epigenetic regulator (*Chaer*) was described to be downregulated in pressure-overload HF. *Chaer* binds to PRC2 components SUZ12 and EZH2 to interfere with the trimethylation of H3K27, a known suppressive chromatin mark, in their target genes. Thereby, *Chaer* prevents the epigenetic repression of CH-related genes, promoting the development of the disease (Wang et al., 2016b).

During an AMI, hypoxia induces the activation of p53 in cardiomyocytes that upregulates maternally expressed gene 3 (*Meg3*) expression (Wu et al., 2018). *MEG3* has been described to act as a scaffold for the PRC2 complex, bringing together EZH2 and JARID2, to exert its epigenetic function in embryonic stem cells (Kaneko et al., 2014), breast cancer (Mondal et al., 2015), and aging endothelium (Boon et al., 2016a). However, in hypoxic cardiomyocytes, *Meg3* works as a decoy for FUS proteins, a factor required for DNA damage repair (Efimova et al., 2017). As result, cardiomyocytes enter apoptosis. This is a clear example of how a lncRNA can have different functions and partners depending on the cell type and the biological context.

One of the contributors to AMI impaired healing is the massive infiltration of immune cells, and it has been described that at least a part of the myeloid cells that migrate to the infarction come from the spleen (Swirski et al., 2009). Interestingly, a lncRNA termed *Lethe* has been shown to be downregulated in the spleen of aged individuals, and its function is the binding of NFκB subunit RelA, interfering with its pro-inflammatory function (Rapicavoli et al., 2013). This could contribute to the impaired healing and therefore worsen AMI recovery in the elderly.

Recently, several lncRNAs, including some that are well described to act through other mechanisms, have been claimed to work as miRNA sponges that prevent their function in RNA interference. For instance, the hypertrophy-related gene *Myd88* is a target of *miR-489*, which is downregulated upon AngII stimulation due to the upregulation of the lncRNA CH-related factor (*Chrf*), which binds to the miRNA counteracting its inhibitory effect on *Myd88* expression (Wang et al., 2014a). It is worth to mention that there is still a controversy about this mechanism for lncRNA function (Thomson and Dinger, 2016). It is unclear how the stoichiometry of these miRNA–lncRNA binding fits, as miRNAs are usually present in much higher copy numbers than lncRNAs in the cell, and most studies only show a single complementary site per lncRNA. In Table 1, we summarize the lncRNAs that have been described as miRNAs sponges in the context of CVD.

**Table 1:** LncRNAs acting as miRNA sponges in the context of CVD.

**Disease**	**lncRNA**	**Reference**	**Sponged miRNA**	**miRNA target**	**Cell type**
CH	*Ror*	Jiang et al. (2016)	*miR-133*	Not studied	CM
CH	*Hotair*	Lai et al. (2017)	*miR-19*	*Pten*	CM
CH	*Plscr4*	Lv et al. (2018)	*miR-214*	*Mfn2*	CM
CH	*Chrf*	Wang et al. (2014a)	*miR-489*	*Myd88*	CM
AMI	*Ftx*	Long et al. (2018)	*miR-29b-1-5p*	*Bcl2l2*	CM
AMI	*Apf*	Wang et al. (2015)	*miR-188-3p*	*Atg7*	CM
AMI	*Carl*	Wang et al. (2014b)	*miR-539*	*Phb2*	CM
AMI	*Nrf*	Wang et al. (2016a)	*miR-873*	*Ripk1/ripk3*	CM
AMI	*Zfas1*	Wu et al. (2017)	*miR-150*	*Crp*	CM
AMI	*Malat1*	Huang et al. (2018)	*miR-145*	*Tgfβ1*	FB
Arrhythmia	*Kcnq1ot1*	Shen et al. (2018)	*miR-384b*	*Cacna1c*	CM
Diabetic HF	*Kcnq1ot1*	Yang et al. (2018)	*miR-214-3p*	*Caspase 1*	CM

CM, cardiomyocytes; FB, fibroblasts.

### Guide lncRNAs

A fibroblast-enriched lncRNA, Wisp2 super-enhancer-associated RNA (*Wisper*), is upregulated after AMI in mice and in patients with aortic stenosis, both diseases that trigger cardiac fibrosis and left ventricular (LV) dysfunction. Indeed, *Wisper* expression correlates with the extent of cardiac fibrosis. Silencing of *Wisper* decreases the expression of profibrotic genes, as *Col1a3* and *Tgfb2*, in cardiac but not lung fibroblasts, while CRISPR-on-mediated *Wisper* induction boosts the expression of these genes. Mice treated with GapmeRs targeting *Wisper* develop much less cardiac fibrosis after AMI induction, and consequently, conserve better LV function. Mechanistically, *Wisper* binds TIAR and retains it in the nucleus, guiding it to bind to and promote processing of lysil hydroxylase 2 mRNA, a mediator of collagen crosslinking (Micheletti et al., 2017).

Another novel mechanism has been described for *Meg3* in cardiac fibroblasts in the development of HFpEF (Piccoli et al., 2017). In a murine model of pressure-overload HF, *Meg3* was downregulated in cardiac fibroblasts in the late stages of cardiac remodeling. Downregulation of *Meg3* repressed expression of genes of the MMP family, specifically *Mmp2*. *Mmp2* is a target of the TGFβ1 pathway and contains a canonical p53-binding site in its promoter. Knockdown of *Meg3 in vivo* prevents *Mmp2* expression, ameliorating fibrosis, and diastolic dysfunction in pressure-overload hearts. The authors propose that, under stress conditions that trigger TGFβ1 pathway activation, *Meg3* binds p53 and directs it to the *Mmp2* promoter, inducing its expression and thereby promoting cardiac fibrosis.

In the previous cases, the lncRNA acted in trans, but lncRNAs can also act in cis. For instance, Modarresi et al. (2012) describe a lncRNA located antisense to *BDNF*, named *BDNF-AS*, that recruits EZH2 to the promoter of *BDNF* to epigenetically repress its expression. In a cardiac context, *Bdnf-as* has been shown to be necessary for *Bdnf* downregulation in an *in vitro* model of ischemia/reperfusion that mediates the response to cellular damage through the VEGF/AKT pathway (Zhao et al., 2017).

A recently described lncRNA involved in telomere protection and cellular senescence is telomeric repeat-containing RNA (*TERRA*). Although telomeres are heterochromatic regions, it has been demonstrated that these noncoding transcripts are transcribed from the subtelomere toward the telomere of several chromosomes. Particularly, the TERRA molecule arising from the q-arm of human chromosome 20 (*20q-TERRA)* has an important role in telomeric heterochromatin assembly, telomere length maintenance, and protection. *TERRA* transcripts recruit the PRC2 complex to the telomere, where it regulates the heterochromatic status of the telomere (Montero et al., 2018). A role for *TERRA* in the cardiovascular system is, however, not yet described.

### Scaffold and enhancer-associated lncRNAs

Although the binding of transcription factors to the promoter is the main determinant to activate the expression of a certain gene, it is important to stress the importance of enhancers in this process. An enhancer is a regulatory sequence in the DNA that lies relatively far away from the transcription start site but dictates the level of expression of its target gene and usually includes chromosomal looping to bring together the different regulatory proteins involved (Ong and Corces, 2011). There is increasing evidence that lncRNAs can act as a scaffold to bind the regulators, transcription factors, DNA enhancer, and promoter sequences in the loop to activate the expression of a gene.

One of the typical features in the onset of HF is the re-expression of the cardiac fetal gene program. The study of the expression of mouse fetal enhancer *mm67*, *mm85*, *mm130*, and *mm132* revealed that they are associated with lncRNAs that are critical for their role in cardiac development and are also expressed in the adult heart. Moreover, RNA expression analysis at 7 days post-AMI showed that lncRNAs *mm67* and *mm85* and their target myocardin were downregulated, while *mm130* and *mm132* were upregulated at 14 days after AMI, which correlated with the expression of their target genes *Tbx20* and *endothelin-1* and reactivation of the cardiac fetal and stress signature markers βMHC, ANF, and BNP. These results were also replicated in a pressure-overload surgical model, proving the importance of enhancer regions in the response to cardiac stress (Ounzain et al., 2014).

In a related publication of the same laboratory, mouse cardiac tissue was isolated 2 weeks after AMI and deep-sequenced to look for novel lncRNAs involved in the signaling cascades that lead to post-infarct HF. The authors found a set of lncRNAs that were differentially regulated compared to sham hearts, cardiac-specific and located in vicinity of genes with roles in cardiac development and transcriptional control, although their expression did not correlate with these. However, it was found that the expression of those lncRNAs was correlated with the appearance of echocardiography-defined physiological traits as ejection fraction or left ventricular diameter. Also, they were associated with active enhancer chromatin states, pointing to an enhancer-associated nature during cardiac precursor cell (CPC) transition to mature cardiomyocytes. For instance, *Novlnc6* is expressed from *mm74*, a cardiac enhancer active in the embryonic left ventricle and correlates with *Bmp10* and *Nkx2.5* expression during cardiac development and in dilated cardiomyopathy patients (Ounzain et al., 2015b).

A special type of enhancers are the so-called super enhancers, which are larger (up to 10 kb long) and are specialized in cell-identity regulation (Hnisz et al., 2013). In the heart, the specification of cell identity is particularly important during cardiac regeneration, when CPCs need to differentiate into mature cardiomyocytes. In a recent publication, profiling of RNA expression in CPCs revealed a novel lncRNA, cardiac mesoderm enhancer-associated ncRNA (*CARMEN*) that is located in an active super enhancer region, is highly expressed during CPC differentiation, and mediates cardiac fate specification. In the adult heart, the three isoforms of *CARMEN* are differentially regulated in patients with dilated cardiomyopathy and CH. This lncRNA exert its function by binding to PRC2 components EZH2 and SUZ12 and recruiting them both in cis and in trans to repress target gene expression (Ounzain et al., 2015a).

### Other lncRNAs regulated in cardiac diseases

Profiling of lncRNAs expressed in cardiac tissue in a pressure-overload mouse model revealed a lncRNA that is upregulated in cardiomyocytes under prohypertrophic stimuli, named CH-associated transcript (*Chast*). *Chast* expression is activated by the CN/NFAT pathway, one of the canonical signaling pathways implicated in hypertrophy (Heineke and Molkentin, 2006). *Chast* gene is located near *Plekhm1* and acts in cis downregulating *Plekhm1* expression, which is necessary to trigger a hypertrophy gene expression profile. Indeed, viral-mediated overexpression of *Chast* in mice induces a spontaneous CH phenotype, while knockdown prevents and decreases CH after pressure-overload. It is unknown what the specific mechanism is that allows *Chast* to interfere with *Plekhm1* expression (Viereck et al., 2016).

Another lncRNA profiling study during aging in cardiac and skeletal muscles showed that the aging process induces differential expression of lncRNAs even in apparently similar tissues. In the heart, four lncRNAs are upregulated specifically in the aging cardiomyocyte. Cluster analysis revealed that this specific expression pattern correlates with negative regulation of myotube differentiation and muscle contraction (Chen et al., 2018). In the same line, Abdelmohsen et al. (2013) performed expression profiling of old versus earlier passage fibroblasts and found out numerous lncRNAs, including the known *MALAT1* and myocardial infarction-associated transcript *(MIAT*) and a novel lncRNA, termed senescence-associated lncRNA 1, that were downregulated during aging. Silencing of any of them triggers a senescent phenotype in cultured fibroblasts and may be of interest in cardiac aging.

A microarray-based RNA expression profiling in hearts of mice subjected to AMI for 24 h shows the upregulation of two novel lncRNAs, myocardial infarction-associated transcript 1 and 2 (*Mirt1* and *Mirt2*). *Mirt1* is expressed in the remote zone of the myocardium by cardiac fibroblasts and correlates with the expression of genes implicated in cardiac remodeling (Zangrando et al., 2014). Knockdown of *Mirt1* partially prevented cardiac dysfunction, apoptosis and monocyte infiltration after AMI through inhibition of the NFκB pathway, although the mechanism is not fully studied (Li et al., 2017).

A study of the lncRNA expression profile of peripheral blood mononuclear cells (PBMNCs) in HF patients allowed for the identification of a novel HF-associated lncRNA, heart disease-associated transcript 2 (*HEAT2*), which is expressed in eosinophils and basophiles but not in other types of immune cells. *HEAT2* was found to promote PBMNC adhesion to the endothelium and transmigration, two important processes in the immune response initiation. *HEAT2* binds to H3K27me3 repressive marks, and it is thought to act by *trans*-regulation of gene expression (Boeckel et al., 2018).

## LncRNAs as biomarkers

The first time that the lncRNA *MIAT* was identified was in a large-scale association study in the Japanese population with the aim to identify single nucleotide polymorphisms (SNPs) in heart tissue that helped predicting a genetic risk for AMI. The authors found a locus containing a SNP with a strong association with AMI. This locus corresponds to a lncRNA that the authors named *MIAT*. Although the function of *MIAT* was not studied, it was suggested that it possibly binds to a transcription factor and increases transcriptional activity in the cell (Ishii et al., 2006). Another attempt of finding SNPs that predict coronary artery disease revealed an association between the risk of atherosclerosis and presence in plasma of different variants of antisense noncoding RNA in the INK4 locus (*ANRIL*) (Liu et al., 2009; Holdt et al., 2010; Yari et al., 2018). *ANRIL* is described to bind two proteins in the PRC1 and PRC2 complexes to induce histone methylation and silencing of the *CDKN2A/B* locus, which is a key for cell survival (Congrains et al., 2013).

In 2014, two different studies in plasma from CVD patients revealed new potential biomarker lncRNAs. Kumarswamy et al. (2014) found that the presence of long intergenic noncoding RNA predicting cardiac remodeling (*LIPCAR*) in plasma of recovering AMI patients was a predictor of adverse cardiac remodeling and HF. On the other side, Vausort et al. (2014) studied the expression profile of five lncRNAs (*aHIF*, *ANRIL*, *KCNQ1OT1*, *MIAT*, and *MALAT1*) in PBMNCs of patients with AMI and found that the last four were significantly downregulated in patients with ST-segment-elevation AMI, and predicted the individuals that would suffer ventricular remodeling, particularly in the case of *ANRIL* and *KCNQ1OT1*. A recent study in the Chinese population has shown two lncRNAs as biomarkers of HF: noncoding repressor of NFAT (*NRON*) and *MHRT*. *NRON* and *MHRT* are upregulated in the plasma of HF patients and correlate with presence of other cardiac biomarkers in blood (Xuan et al., 2017).

## Conclusion

The advances in healthcare have increased the lifespan in the Western societies, but life quality in the elderly is still lagging due to the high prevalence of cardiac diseases. There is increasing evidence for the role of lncRNAs in CVDs and aging, including their potential as biomarkers of worsening prognosis after cardiac events. However, the field is still too novel to bring a therapeutic option for patients, and more profound studies are required, as well as a critical view of the discipline to completely and verifiably understand the molecular mechanisms that define lncRNA function in the different cell subsets and under different pathological contexts.

## References

[ref1] Abdelmohsen K., Panda A., Kang M.J., et al. (2013). Senescence-associated lncRNAs: senescence-associated long noncoding RNAs. Aging Cell 12, 890–900. 23758631 10.1111/acel.12115 PMC3773026

[ref2] Bar C., Bernardes de Jesus B., Serrano R., et al. (2014). Telomerase expression confers cardioprotection in the adult mouse heart after acute myocardial infarction. Nat. Commun. 5, 5863. 25519492 10.1038/ncomms6863 PMC4871230

[ref3] Boeckel J.N., Perret M.F., Glaser S.F., et al. (2018). Identification and regulation of the long non-coding RNA Heat2 in heart failure. J. Mol. Cell. Cardiol. 126, 13–22. 30445017 10.1016/j.yjmcc.2018.11.004

[ref4] Boon R.A., Hofmann P., Michalik K.M., et al. (2016a). Long noncoding RNA Meg3 controls endothelial cell aging and function: implications for regenerative angiogenesis. J. Am. Coll. Cardiol. 68, 2589–2591. 27931619 10.1016/j.jacc.2016.09.949

[ref5] Boon R.A., Iekushi K., Lechner S., et al. (2013). MicroRNA-34a regulates cardiac ageing and function. Nature 495, 107–110. 23426265 10.1038/nature11919

[ref6] Boon R.A., Jae N., Holdt L., et al. (2016b). Long noncoding RNAs: from clinical genetics to therapeutic targets? J. Am. Coll. Cardiol. 67, 1214–1226. 26965544 10.1016/j.jacc.2015.12.051

[ref7] Bootman M.D., Smyrnias I., Thul R., et al. (2011). Atrial cardiomyocyte calcium signalling. Biochim. Biophys. Acta 1813, 922–934. 21295621 10.1016/j.bbamcr.2011.01.030

[ref8] Calado R.T., and Young N.S. (2009). Telomere diseases. N. Engl. J. Med. 361, 2353–2365. 20007561 10.1056/NEJMra0903373 PMC3401586

[ref9] Chen J., Zou Q., Lv D., et al. (2018). Comprehensive transcriptional landscape of porcine cardiac and skeletal muscles reveals differences of aging. Oncotarget 9, 1524–1541. 29416711 10.18632/oncotarget.23290 PMC5788579

[ref10] Christensen K., Doblhammer G., Rau R., et al. (2009). Ageing populations: the challenges ahead. Lancet 374, 1196–1208. 19801098 10.1016/S0140-6736(09)61460-4 PMC2810516

[ref11] Congrains A., Kamide K., Ohishi M., et al. (2013). ANRIL: molecular mechanisms and implications in human health. Int. J. Mol. Sci. 14, 1278–1292. 23306151 10.3390/ijms14011278 PMC3565320

[ref12] Devaux Y., Zangrando J., Schroen B., et al. (2015). Long noncoding RNAs in cardiac development and ageing. Nat. Rev. Cardiol. 12, 415–425. 25855606 10.1038/nrcardio.2015.55

[ref13] Efimova A.D., Ovchinnikov R.K., Roman A.Y., et al. (2017). The FUS protein: physiological functions and a role in amyotrophic lateral sclerosis. Mol. Biol. 51, 387–399. 10.7868/S0026898417020094 28707655

[ref14] Feridooni H.A., Dibb K.M., and Howlett S.E. (2015). How cardiomyocyte excitation, calcium release and contraction become altered with age. J. Mol. Cell. Cardiol. 83, 62–72. 25498213 10.1016/j.yjmcc.2014.12.004

[ref15] Fleg J.L., Forman D.E., Berra K., et al. (2013). Secondary prevention of atherosclerotic cardiovascular disease in older adults: a scientific statement from the American Heart Association. Circulation 128, 2422–2446. 24166575 10.1161/01.cir.0000436752.99896.22 PMC4171129

[ref16] Frangogiannis N.G. (2015). Pathophysiology of myocardial infarction. Compr. Physiol. 5, 1841–1875. 26426469 10.1002/cphy.c150006

[ref17] Frank S., Aguirre A., Hescheler J., et al. (2016). A lncRNA perspective into (re)building the heart. Front. Cell Dev. Biol. 4, 128. 27882316 10.3389/fcell.2016.00128 PMC5101577

[ref18] Frankish A., Diekhans M., Ferreira A.M., et al. (2019). GENCODE reference annotation for the human and mouse genomes. Nucleic Acids Res. 47, D766–D773. 30357393 10.1093/nar/gky955 PMC6323946

[ref19] Frey N., and Olson E.N. (2003). Cardiac hypertrophy: the good, the bad, and the ugly. Annu. Rev. Physiol. 65, 45–79. 12524460 10.1146/annurev.physiol.65.092101.142243

[ref20] Gaeta M., Campanella F., Gentile L., et al. (2017). European cardiovascular mortality over the last three decades: evaluation of time trends, forecasts for 2016. Ann. Ig. 29, 206–217. 28383612 10.7416/ai.2017.2148

[ref21] Greco C.M., and Condorelli G. (2015). Epigenetic modifications and noncoding RNAs in cardiac hypertrophy and failure. Nat. Rev. Cardiol. 12, 488–497. 25962978 10.1038/nrcardio.2015.71

[ref22] Han P., and Chang C.P. (2015). Long non-coding RNA and chromatin remodeling. RNA Biol. 12, 1094–1098. 26177256 10.1080/15476286.2015.1063770 PMC4829272

[ref23] Han P., Li W., Lin C.H., et al. (2014). A long noncoding RNA protects the heart from pathological hypertrophy. Nature 514, 102–106. 25119045 10.1038/nature13596 PMC4184960

[ref24] Heidenreich P.A., Trogdon J.G., Khavjou O.A., et al. (2011). Forecasting the future of cardiovascular disease in the United States: a policy statement from the American Heart Association. Circulation 123, 933–944. 21262990 10.1161/CIR.0b013e31820a55f5

[ref25] Heineke J., and Molkentin J.D. (2006). Regulation of cardiac hypertrophy by intracellular signalling pathways. Nat. Rev. Mol. Cell Biol. 7, 589–600. 16936699 10.1038/nrm1983

[ref26] Hermans-Beijnsberger S., Bilsen M. van, and Schroen B. (2018). Long non-coding RNAs in the failing heart and vasculature. Noncoding RNA Res. 3, 118–130. 30175285 10.1016/j.ncrna.2018.04.002 PMC6114261

[ref27] Hnisz D., Abraham B.J., Lee T.I., et al. (2013). Super-enhancers in the control of cell identity and disease. Cell 155, 934–947. 24119843 10.1016/j.cell.2013.09.053 PMC3841062

[ref28] Holdt L.M., Beutner F., Scholz M., et al. (2010). ANRIL expression is associated with atherosclerosis risk at chromosome 9p21. Arterioscler. Thromb. Vasc. Biol. 30, 620–627. 20056914 10.1161/ATVBAHA.109.196832

[ref29] Huang S., Zhang L., Song J., et al. (2018). Long noncoding RNA MALAT1 mediates cardiac fibrosis in experimental postinfarct myocardium mice model. J. Cell. Physiol. 234, 2997–3006. 30146700 10.1002/jcp.27117

[ref30] Ishii N., Ozaki K., Sato H., et al. (2006). Identification of a novel non-coding RNA, MIAT, that confers risk of myocardial infarction. J. Hum. Genet. 51, 1087–1099. 17066261 10.1007/s10038-006-0070-9

[ref31] Jiang F., Zhou X., and Huang J. (2016). Long non-coding RNA-ROR mediates the reprogramming in cardiac hypertrophy. PLoS One 11, e0152767. 27082978 10.1371/journal.pone.0152767 PMC4833345

[ref32] Kaneko , Bonasio R., Saldana-Meyer R., et al. (2014). Interactions between JARID2 and noncoding RNAs regulate PRC2 recruitment to chromatin. Mol. Cell 53, 290–300. 24374312 10.1016/j.molcel.2013.11.012 PMC4026005

[ref33] Kim J., Kim K.M., Noh J.H., et al. (2016). Long noncoding RNAs in diseases of aging. Biochim. Biophys. Acta 1859, 209–221. 26141605 10.1016/j.bbagrm.2015.06.013 PMC4698248

[ref34] Kornfeld O.S., Hwang S., Disatnik M.H., et al. (2015). Mitochondrial reactive oxygen species at the heart of the matter: new therapeutic approaches for cardiovascular diseases. Circ. Res. 116, 1783–1799. 25999419 10.1161/CIRCRESAHA.116.305432 PMC4443500

[ref35] Kovacic J.C., Moreno P., Hachinski V., et al. (2011a). Cellular senescence, vascular disease, and aging: part 1 of a 2-part review. Circulation 123, 1650–1660. 21502583 10.1161/CIRCULATIONAHA.110.007021

[ref36] Kovacic J.C., Moreno P., Nabel E.G., et al. (2011b). Cellular senescence, vascular disease, and aging: part 2 of a 2-part review: clinical vascular disease in the elderly. Circulation 123, 1900–1910. 21537006 10.1161/CIRCULATIONAHA.110.009118

[ref37] Kumarswamy R., Bauters C., Volkmann I., et al. (2014). Circulating long noncoding RNA, LIPCAR, predicts survival in patients with heart failure. Circ. Res. 114, 1569–1575. 24663402 10.1161/CIRCRESAHA.114.303915

[ref38] Lai Y., He S., Ma L., et al. (2017). HOTAIR functions as a competing endogenous RNA to regulate PTEN expression by inhibiting miR-19 in cardiac hypertrophy. Mol. Cell. Biochem. 432, 179–187. 28316060 10.1007/s11010-017-3008-y

[ref39] Lakatta E.G., and Levy D. (2003a). Arterial and cardiac aging: major shareholders in cardiovascular disease enterprises: part I: aging arteries: a ‘set up’ for vascular disease. Circulation 107, 139–146. 12515756 10.1161/01.cir.0000048892.83521.58

[ref40] Lakatta E.G., and Levy D. (2003b). Arterial and cardiac aging: major shareholders in cardiovascular disease enterprises: part II: the aging heart in health: links to heart disease. Circulation 107, 346–354. 12538439 10.1161/01.cir.0000048893.62841.f7

[ref41] Leri A., Franco S., Zacheo A., et al. (2003). Ablation of telomerase and telomere loss leads to cardiac dilatation and heart failure associated with p53 upregulation. EMBO J. 22, 131–139. 12505991 10.1093/emboj/cdg013 PMC140062

[ref42] Li X., Zhou J., and Huang K. (2017). Inhibition of the lncRNA Mirt1 attenuates acute myocardial infarction by suppressing NF-κB activation. Cell. Physiol. Biochem. 42, 1153–1164. 28668956 10.1159/000478870

[ref43] Ling L.H., Kistler P.M., Kalman J.M., et al. (2016). Comorbidity of atrial fibrillation and heart failure. Nat. Rev. Cardiol. 13, 131–147. 26658575 10.1038/nrcardio.2015.191

[ref44] Liu Y., Sanoff H.K., Cho H., et al. (2009). INK4/ARF transcript expression is associated with chromosome 9p21 variants linked to atherosclerosis. PLoS One 4, e5027. 19343170 10.1371/journal.pone.0005027 PMC2660422

[ref45] Long B., Li N., Xu X.X., et al. (2018). Long noncoding RNA FTX regulates cardiomyocyte apoptosis by targeting miR-29b-1-5p and Bcl2l2. Biochem. Biophys. Res. Commun. 495, 312–318. 29117536 10.1016/j.bbrc.2017.11.030

[ref46] Lopez-Otin C., Blasco M.A., Partridge L., et al. (2013). The hallmarks of aging. Cell 153, 1194–1217. 23746838 10.1016/j.cell.2013.05.039 PMC3836174

[ref47] Lv L., Li T., Li X., et al. (2018). The lncRNA Plscr4 controls cardiac hypertrophy by regulating miR-214. Mol. Ther. Nucleic Acids 10, 387–397. 29499950 10.1016/j.omtn.2017.12.018 PMC5862136

[ref48] Maass P.G., Luft F.C., and Bahring S. (2014). Long non-coding RNA in health and disease. J. Mol. Med. 92, 337–346. 24531795 10.1007/s00109-014-1131-8

[ref49] Malvezzi M., Carioli G., Bertuccio P., et al. (2018). Relation between mortality trends of cardiovascular diseases and selected cancers in the European Union, in 1970–2017. Focus on cohort and period effects. Eur. J. Cancer 103, 341–355. 30029971 10.1016/j.ejca.2018.06.018

[ref50] Mao Y.S., Sunwoo H., Zhang B., et al. (2011). Direct visualization of the co-transcriptional assembly of a nuclear body by noncoding RNAs. Nat. Cell Biol. 13, 95–101. 21170033 10.1038/ncb2140 PMC3007124

[ref51] Mathiyalagan P., Keating S.T., Du X.J., et al. (2014). Chromatin modifications remodel cardiac gene expression. Cardiovasc. Res. 103, 7–16. 24812277 10.1093/cvr/cvu122

[ref52] Mercer T.R., and Mattick J.S. (2013). Structure and function of long noncoding RNAs in epigenetic regulation. Nat. Struct. Mol. Biol. 20, 300–307. 23463315 10.1038/nsmb.2480

[ref53] Micheletti R., Plaisance I., Abraham B.J., et al. (2017). The long noncoding RNA Wisper controls cardiac fibrosis and remodeling. Sci. Transl. Med. 9, pii: eaai9118. 10.1126/scitranslmed.aai9118 PMC5643582 28637928

[ref100] Militello G., Hosen M.R., Ponomareva Y., et al. (2018). A novel long non-coding RNA Myolinc regulates myogenesis through TDP-43 and Filip1. J. Mol. Cell Biol. 10, 102–117. 29618024 10.1093/jmcb/mjy025 PMC7191624

[ref54] Miyata S., Minobe W., Bristow M.R., et al. (2000). Myosin heavy chain isoform expression in the failing and nonfailing human heart. Circ. Res. 86, 386–390. 10700442 10.1161/01.res.86.4.386

[ref55] Modarresi F., Faghihi M.A., Lopez-Toledano M.A., et al. (2012). Inhibition of natural antisense transcripts in vivo results in gene-specific transcriptional upregulation. Nat. Biotechnol. 30, 453–459. 22446693 10.1038/nbt.2158 PMC4144683

[ref56] Mondal T., Subhash S., Vaid R., et al. (2015). MEG3 long noncoding RNA regulates the TGF-β pathway genes through formation of RNA–DNA triplex structures. Nat. Commun. 6, 7743. 26205790 10.1038/ncomms8743 PMC4525211

[ref57] Montero J.J., Lopez-Silanes I., Megias D., et al. (2018). TERRA recruitment of polycomb to telomeres is essential for histone trymethylation marks at telomeric heterochromatin. Nat. Commun. 9, 1548. 29670078 10.1038/s41467-018-03916-3 PMC5906467

[ref58] North B.J., and Sinclair D.A. (2012). The intersection between aging and cardiovascular disease. Circ. Res. 110, 1097–1108. 22499900 10.1161/CIRCRESAHA.111.246876 PMC3366686

[ref59] Okazaki Y., Furuno M., Kasukawa T., et al. (2002). Analysis of the mouse transcriptome based on functional annotation of 60,770 full-length cDNAs. Nature 420, 563–573. 12466851 10.1038/nature01266

[ref60] Ong C.T., and Corces V.G. (2011). Enhancer function: new insights into the regulation of tissue-specific gene expression. Nat. Rev. Genet. 12, 283–293. 21358745 10.1038/nrg2957 PMC3175006

[ref61] Ounzain S., Micheletti R., Arnan C., et al. (2015a). CARMEN, a human super enhancer-associated long noncoding RNA controlling cardiac specification, differentiation and homeostasis. J. Mol. Cell. Cardiol. 89, 98–112. 26423156 10.1016/j.yjmcc.2015.09.016

[ref62] Ounzain S., Micheletti R., Beckmann T., et al. (2015b). Genome-wide profiling of the cardiac transcriptome after myocardial infarction identifies novel heart-specific long non-coding RNAs. Eur. Heart J. 36, 353–368a. 24786300 10.1093/eurheartj/ehu180 PMC4320320

[ref63] Ounzain S., Pezzuto I., Micheletti R., et al. (2014). Functional importance of cardiac enhancer-associated noncoding RNAs in heart development and disease. J. Mol. Cell. Cardiol. 76, 55–70. 25149110 10.1016/j.yjmcc.2014.08.009 PMC4445080

[ref64] Owan T.E., and Redfield M.M. (2005). Epidemiology of diastolic heart failure. Prog. Cardiovasc. Dis. 47, 320–332. 16003647 10.1016/j.pcad.2005.02.010

[ref65] Paneni F., Diaz Canestro C., Libby P., et al. (2017). The aging cardiovascular system: understanding it at the cellular and clinical Levels. J. Am. Coll. Cardiol. 69, 1952–1967. 28408026 10.1016/j.jacc.2017.01.064

[ref66] Piccoli M.T., Gupta S.K., Viereck J., et al. (2017). Inhibition of the cardiac fibroblast-enriched lncRNA Meg3 prevents cardiac fibrosis and diastolic dysfunction. Circ. Res. 121, 575–583. 28630135 10.1161/CIRCRESAHA.117.310624

[ref67] Quinodoz S., and Guttman M. (2014). Long noncoding RNAs: an emerging link between gene regulation and nuclear organization. Trends Cell Biol. 24, 651–663. 25441720 10.1016/j.tcb.2014.08.009 PMC4254690

[ref68] Rapicavoli N.A., Qu K., Zhang J., et al. (2013). A mammalian pseudogene lncRNA at the interface of inflammation and anti-inflammatory therapeutics. eLife 2, e00762. 23898399 10.7554/eLife.00762 PMC3721235

[ref69] Redfield M.M. (2016). Heart failure with preserved ejection fraction. N. Engl. J. Med. 375, 1868–1877. 27959663 10.1056/NEJMcp1511175

[ref70] Sahin E., Colla S., Liesa M., et al. (2011). Telomere dysfunction induces metabolic and mitochondrial compromise. Nature 470, 359–365. 21307849 10.1038/nature09787 PMC3741661

[ref71] Saltzman H.E. (2014). Arrhythmias and heart failure. Cardiol. Clin. 32, 125–133. 24286583 10.1016/j.ccl.2013.09.005

[ref72] Schmitt A.M., Garcia J.T., Hung T., et al. (2016). An inducible long noncoding RNA amplifies DNA damage signaling. Nat. Genet. 48, 1370–1376. 27668660 10.1038/ng.3673 PMC5083181

[ref73] Sharifi-Sanjani M., Oyster N.M., Tichy E.D., et al. (2017). Cardiomyocyte-specific telomere shortening is a distinct signature of heart failure in humans. J. Am. Heart Assoc. 6, pii: e005086. 10.1161/JAHA.116.005086 PMC5634248 28882819

[ref74] Shen C., Kong B., Liu Y., et al. (2018). YY1-induced upregulation of lncRNA KCNQ1OT1 regulates angiotensin II-induced atrial fibrillation by modulating miR-384b/CACNA1C axis. Biochem. Biophys. Res. Commun. 505, 134–140. 30241939 10.1016/j.bbrc.2018.09.064

[ref75] Staerk L., Wang B., Lunetta K.L., et al. (2017). Association between leukocyte telomere length and the risk of incident atrial fibrillation: the Framingham heart study. J. Am. Heart Assoc. 6, pii: e006541. 10.1161/JAHA.117.006541 PMC5721755 29138179

[ref76] Swirski F.K., Nahrendorf M., Etzrodt M., et al. (2009). Identification of splenic reservoir monocytes and their deployment to inflammatory sites. Science 325, 612–616. 19644120 10.1126/science.1175202 PMC2803111

[ref77] Taft R.J., Pang K.C., Mercer T.R., et al. (2010). Non-coding RNAs: regulators of disease. J. Pathol. 220, 126–139. 19882673 10.1002/path.2638

[ref78] Thomson D.W., and Dinger M.E. (2016). Endogenous microRNA sponges: evidence and controversy. Nat. Rev. Genet. 17, 272–283. 27040487 10.1038/nrg.2016.20

[ref79] Uchida S., and Dimmeler S. (2015). Long noncoding RNAs in cardiovascular diseases. Circ. Res. 116, 737–750. 25677520 10.1161/CIRCRESAHA.116.302521

[ref80] Ulitsky I., Shkumatava A., Jan C.H., et al. (2011). Conserved function of lincRNAs in vertebrate embryonic development despite rapid sequence evolution. Cell 147, 1537–1550. 22196729 10.1016/j.cell.2011.11.055 PMC3376356

[ref81] Berlo J.H. van, Maillet M., and Molkentin J.D. (2013). Signaling effectors underlying pathologic growth and remodeling of the heart. J. Clin. Invest. 123, 37–45. 23281408 10.1172/JCI62839 PMC3533272

[ref82] Vausort M., Wagner D.R., and Devaux Y. (2014). Long noncoding RNAs in patients with acute myocardial infarction. Circ. Res. 115, 668–677. 25035150 10.1161/CIRCRESAHA.115.303836

[ref83] Viereck J., Kumarswamy R., Foinquinos A., et al. (2016). Long noncoding RNA Chast promotes cardiac remodeling. Sci. Transl. Med. 8, 326ra322. 10.1126/scitranslmed.aaf1475 26888430

[ref84] Vigen R., Maddox T.M., and Allen L.A. (2012). Aging of the United States population: impact on heart failure. Curr. Heart Fail. Rep. 9, 369–374. 22940871 10.1007/s11897-012-0114-8 PMC3893701

[ref85] Wang K., Liu C.Y., Zhou L.Y., et al. (2015). APF lncRNA regulates autophagy and myocardial infarction by targeting miR-188-3p. Nat. Commun. 6, 6779. 25858075 10.1038/ncomms7779

[ref86] Wang K., Liu F., Liu C.Y., et al. (2016a). The long noncoding RNA NRF regulates programmed necrosis and myocardial injury during ischemia and reperfusion by targeting miR-873. Cell Death Differ. 23, 1394–1405. 27258785 10.1038/cdd.2016.28 PMC4947670

[ref87] Wang K., Liu F., Zhou L.Y., et al. (2014a). The long noncoding RNA CHRF regulates cardiac hypertrophy by targeting miR-489. Circ. Res. 114, 1377–1388. 24557880 10.1161/CIRCRESAHA.114.302476

[ref88] Wang K., Long B., Zhou L.Y., et al. (2014b). CARL lncRNA inhibits anoxia-induced mitochondrial fission and apoptosis in cardiomyocytes by impairing miR-539-dependent PHB2 downregulation. Nat. Commun. 5, 3596. 24710105 10.1038/ncomms4596

[ref89] Wang K.C., and Chang H.Y. (2011). Molecular mechanisms of long noncoding RNAs. Mol. Cell 43, 904–914. 21925379 10.1016/j.molcel.2011.08.018 PMC3199020

[ref90] Wang Z., Zhang X.J., Ji Y.X., et al. (2016b). The long noncoding RNA Chaer defines an epigenetic checkpoint in cardiac hypertrophy. Nat. Med. 22, 1131–1139. 27618650 10.1038/nm.4179 PMC5053883

[ref91] Wellenius G.A., and Mittleman M.A. (2008). Disparities in myocardial infarction case fatality rates among the elderly: the 20-year Medicare experience. Am. Heart J. 156, 483–490. 18760130 10.1016/j.ahj.2008.04.009 PMC2574015

[ref92] Williams M.A., Fleg J.L., Ades P.A., et al. (2002). Secondary prevention of coronary heart disease in the elderly (with emphasis on patients > or =75 years of age): an American Heart Association scientific statement from the council on clinical cardiology subcommittee on exercise, cardiac rehabilitation, and prevention. Circulation 105, 1735–1743. 11940556 10.1161/01.cir.0000013074.73995.6c

[ref93] Wu H., Zhao Z.A., Liu J., et al. (2018). Long noncoding RNA Meg3 regulates cardiomyocyte apoptosis in myocardial infarction. Gene Ther. 25, 511–523. 30287867 10.1038/s41434-018-0045-4

[ref94] Wu T., Wu D., Wu Q., et al. (2017). Knockdown of Long non-coding RNA-ZFAS1 protects cardiomyocytes against acute myocardial infarction via anti-apoptosis by regulating miR-150/CRP. J. Cell. Biochem. 118, 3281–3289. 28295592 10.1002/jcb.25979

[ref95] Xuan L., Sun L., Zhang Y., et al. (2017). Circulating long non-coding RNAs NRON and MHRT as novel predictive biomarkers of heart failure. J. Cell. Mol. Med. 21, 1803–1814. 28296001 10.1111/jcmm.13101 PMC5571539

[ref96] Yang F., Qin Y., Wang Y., et al. (2018). LncRNA KCNQ1OT1 mediates pyroptosis in diabetic cardiomyopathy. Cell. Physiol. Biochem. 50, 1230–1244. 30355944 10.1159/000494576

[ref97] Yari M., Bitarafan S., Broumand M.A., et al. (2018). Association between Long noncoding RNA ANRIL expression variants and susceptibility to coronary artery disease. Int. J. Mol. Cell. Med. 7, 1–7. 30234067 10.22088/IJMCM.BUMS.7.1.1 PMC6134424

[ref98] Zangrando J., Zhang L., Vausort M., et al. (2014). Identification of candidate long non-coding RNAs in response to myocardial infarction. BMC Genomics 15, 460. 24917243 10.1186/1471-2164-15-460 PMC4070571

[ref99] Zhao R., Wang X., Wang H., et al. (2017). Inhibition of long noncoding RNA BDNF-AS rescues cell death and apoptosis in hypoxia/reoxygenation damaged murine cardiomyocyte. Biochimie 138, 43–49. 28363791 10.1016/j.biochi.2017.03.018

